# Delayed Diagnosis of Buried Bumper Syndrome When Only the Jejunostomy Extension is Used in a Percutaneous Endoscopic Gastrostomy-jejunostomy Levodopa-carbidopa Intestinal Gel Delivery System

**DOI:** 10.7759/cureus.4568

**Published:** 2019-04-30

**Authors:** Ahmad A Abu-Heija, Maher Tama, Usama Abu-Heija, Mowyad Khalid, Omar Al-Subee

**Affiliations:** 1 Internal Medicine, Wayne State University, Detroit Medical Center, Detroit, USA; 2 Gastroenterology, Wayne State University, Detroit Medical Center, Detroit, USA; 3 Miscellaneous, Jordan University of Science and Technology, Amman, JOR; 4 Gastroenterology, McLaren Hospital, Flint, USA

**Keywords:** gastrostomy, carbidopa-levodopa, parkinson disease, infusion pumps, drug delivery systems, buried bumper syndrome, peg-j complication

## Abstract

Direct intrajejunal levodopa-carbidopa intestinal gel (LCIG) administered through a percutaneous endoscopic gastrostomy (PEG) with a jejunal extension tube (PEG-J) is an FDA-approved modality for treatment of patients with advanced Parkinson’s disease (PD). Buried bumper syndrome (BBS) is a rare complication associated with PEG tubes inserted for drug administration or enteral feeding. The syndrome is diagnosed endoscopically revealing burial of the internal bumper in the gastric wall, causing numerous serious complications. When only the J extension of a PEG-J is used to deliver medications, and the G tube is not utilized, a delay in the diagnosis of BBS can occur. This is likely as the small caliber J extension tube remains patent and functional. We present the case of an elderly patient with advanced PD on LCIG therapy, who presented with a dislodged J-tube from a PEG-J system. Endoscopy revealed BBS that had likely developed prior to dislodgment of J-extension and despite a conservative approach, the internal bumper needed to be surgically extracted to prevent further complications.

## Introduction

Percutaneous endoscopic gastrostomy (PEG) tubes have seen an increasing use globally in the last few decades as means for enteral nutrition, drug delivery, and gastric decompression, with estimates of an excess of 250,000 PEG tubes inserted annually in the United States alone [1]. Complications associated with PEG tubes arise frequently. Nevertheless, improvements in technique and post-insertion site care have diminished adverse events (AEs) and increased the longevity of these systems. Buried bumper syndrome (BBS) is a rare complication associated with PEG tube placement, with an approximate incidence of 0.3%-2.4% [1-2]. BBS refers to the displacement of the internal gastrostomy bumper into the PEG tract. The internal bumper can be found covered by gastric mucosa endoscopically, completely buried in the gastric wall, and in severe cases traversing the stoma tract and found near the skin surface. Presentation depends on the time lag between insertion and diagnosis, as well as the technique utilized during placement. Excessive traction on an overtly tightened external PEG bumper is postulated to be the etiology behind BBS; other factors such as weight gain, chronic cough, and improper post-insertion PEG tube care can contribute to the presentation [2-3].

Levodopa remains the mainstay of pharmacotherapy for managing motor symptoms associated with advanced Parkinson’s disease (PD) [4]. Levodopa is mainly absorbed in the proximal small intestine, thus direct intrajejunal infusion delivered continuously prevents large fluctuations in the serum drug concentration seen in conventionally administered oral levodopa, especially observed with delayed gastric emptying found in this patient population [5-6]. Hence, the increased utility of a new drug delivery system marketed in the United States as Duopa (AbbVie™, North Chicago, IL); the drug is administered through a PEG with a jejunal extension tube (J-tube), also known as a PEG-J system. The system allows for continuous direct intrajejunal levodopa-carbidopa intestinal gel (LCIG) delivery, via a portable infusion pump connected to the J-tube. A multitude of complications is associated with the use of the device [5-7].

## Case presentation

**Figure 1:**
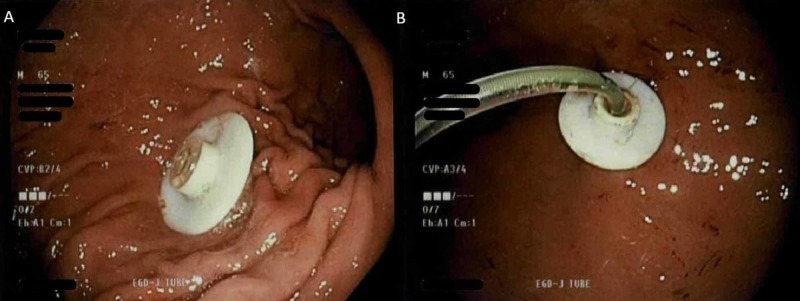
(A) Successful insertion of the PEG tube, followed by (B) insertion of a jejunal extension tube (J-tube) under fluoroscopic guidance, two months prior to presentation. PEG: Percutaneous endoscopic gastrostomy.

We present the case of a 65-year-old male with advanced PD who presented after accidental dislodgement of the jejunostomy tube (J-tube) from the PEG site a day prior to admission. Two months prior, the patient had undergone successful PEG tube placement (Figure 1A) and the introduction of a trans-gastric jejunostomy tube extension (Figure 1B) for direct jejunal administration of LCIG. The PEG-J system insertion was complicated by peri-stomal cellulitis requiring intravenous antibiotics one week after insertion. Prior to the dislodgement of the J extension, the medication delivery system was completely functional. On presentation, the patient was hemodynamically stable. Abdominal examination revealed the PEG tube in place, however, the J-tube was dislodged completely via the Y-connector externally as witnessed by the patient.

**Figure 2:**
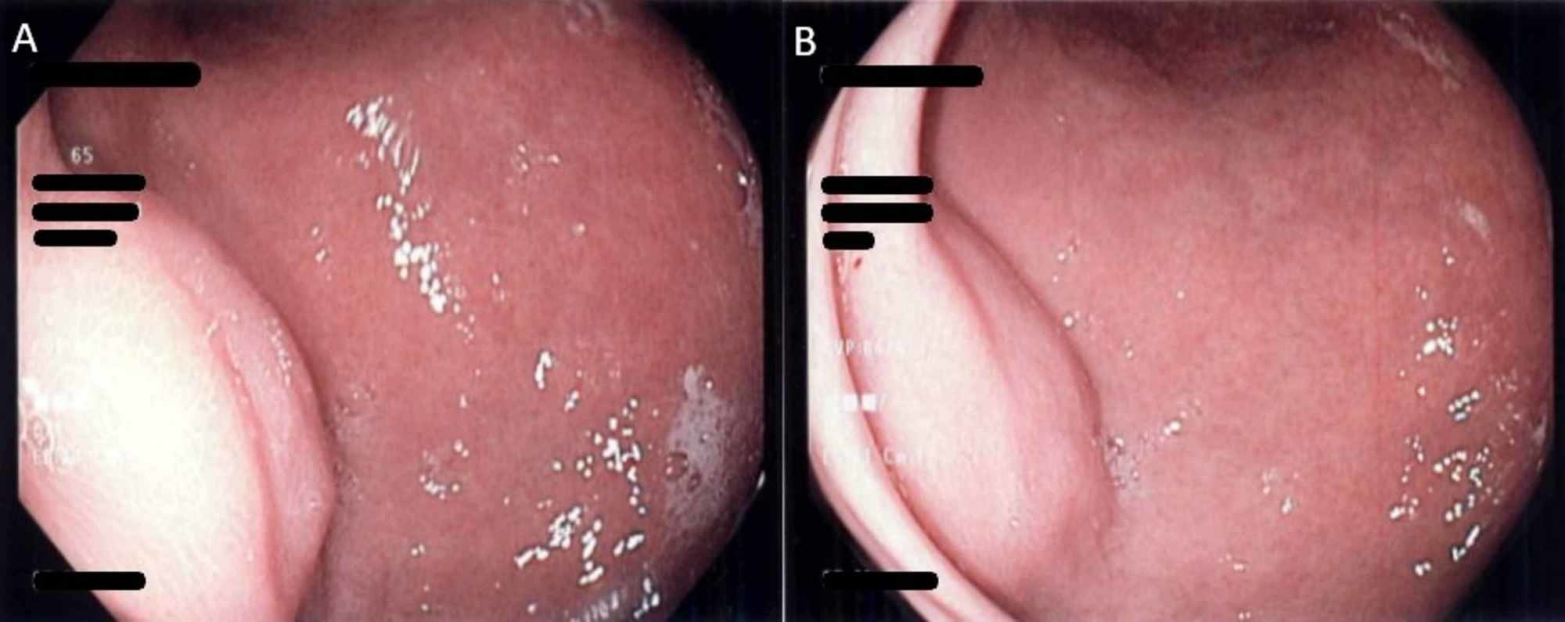
(A & B) Buried bumper syndrome on presentation; the internal bumper completely embedded within the gastric wall, resembling a submucosal mass.

Endoscopic evaluation immediately ensued, revealing complete burial of the internal PEG bumper in the gastric mucosa (Figure 2A, 2B). Due to the typical presentation of our patient and the eventual necessity to remove the internal bumper, we did not utilize the use of endoscopic ultrasound in our patient. Attempts were made to extract the internal bumper via the skin by pulling on the tube externally. However, efforts were unsuccessful and the patient was referred to surgery. Successful surgical extraction of the internal bumper was performed by careful dissection of the open abdominal wall wound and dissecting scar tissue neighboring the internal bumper.

**Figure 3:**
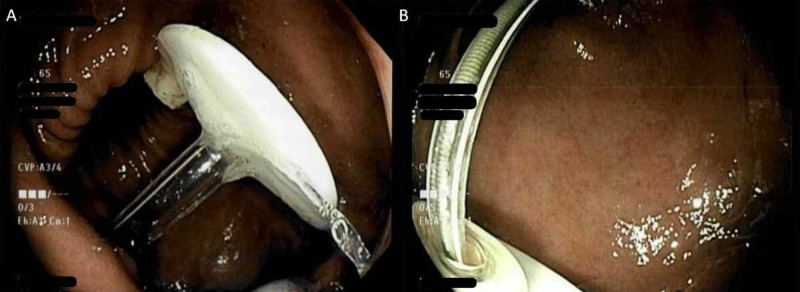
(A) Successful replacement of the PEG tube, and (B) insertion of the jejunal extension tube (J-tube) under fluoroscopic guidance. PEG: Percutaneous endoscopic gastrostomy.

On the following day, and due to the necessity of resuming LCIG, a new PEG-J was inserted endoscopically through the existing open wound in the anterior abdominal wall (Figure 3A, 3B). Suitable resources were given to the patient and his caregiver, with instructions pertaining to adequate PEG-J care and a home nurse was scheduled to visit the patient to provide continuous optimal care. LCIG administration was continued and the patient was seen on follow-up after four weeks and at six months, with sustained normal functioning of the new PEG-J system.

## Discussion

Long-term treatment with LCIG significantly improves motor symptoms of PD and quality of life [8]. AEs related to LCIG therapy via PEG-J have been reported and are mostly related to device insertion and complications related to the tube system. Abdominal pain is the most common AE related to device insertion, affecting approximately a third of patients [6-7]. AEs related to the tubing system, including tube dislodgement, knot formation, kinking, or obstruction are reported in 8-55% of patients [5,7,9]. Gastrostomy site AEs, including incision site infection, erythema, or excessive granulation tissue are also reported in varying degrees in 4-59% of patients, with only one previous case of BBS [5,7,9-10].

BBS is diagnosed endoscopically, with raised clinical suspicion in cases of resistance to pushing the tube internally or resistance to rotation of the tube externally. Endoscopic examination can initially reveal pressure ulceration beneath the internal bumper, followed by mucosal tissue encroaching and eventually covering the internal bumper. A delay in diagnosis can allow for further outward displacement of the internal bumper, that can be complicated by peritonitis, perforation, and formation of an abscess or a phlegmon [1,3]. Also, the cellulitis our patient developed soon after the initial placement likely contributed to edema and swelling extending beyond the boundaries of the internal bumper to later cover it completely.

Management of BBS should be individualized, due to lack of consensus on a standardized approach to this rare complication [1,11-12]. Kejariwal et al. described a “cut and leave it alone” approach in a case series of patients deemed poor surgical candidates, where the PEG-tubes were left in situ and follow-up over 18 months revealed no late complications [11]. A “push and pull inside” approach is described in case series, where the buried bumper is stiffened and pushed inside the stomach after metallic dilators are used to dilate the stoma, followed by endoscopic PEG extraction [1]. Müller-Gerbes et al. described removal via the introduction of a papillotome into the stomach through the severed PEG externally, and through dissection of the adjacent fibrous tissue and tract dilatation, the bumper is pushed into the stomach and retrieved endoscopically [13]. In addition, external traction has been reported to be successful in removing the buried internal bumper [12]. Nevertheless, surgical removal is occasionally needed after the failure of more conservative modalities [1,12].

Proper positioning of the external bumper at the time of PEG-J insertion is of the utmost importance in preventing BBS, with emphasis on leaving approximately 1 cm of space between the external bumper and the skin on insertion [1,3,12]. We believe that there is an underestimation of the true incidence of BBS due to the preserved functionality of the J port even when the internal bumper is completely buried in the gastric wall. Especially that patients using PEG-J for LCIG only need the J extension and do not typically require the use of the G-tube. As such, we recommend frequent examination of the PEG-J system post placement assuring the tube is minimally mobile and not tethered to the underlying deep tissue and we propose the continued use of the G-tube for twice daily water flushes to assure patency of the system and to avoid a late diagnosis of BBS.

## Conclusions

PEG tubes with J-extension (PEG-J) are being increasingly utilized for enteral feeding and administration of LCIG. Most patients using PEG-J for LCIG only need the J extension and do not typically require the use of the G tube of the system. BBS remains a rare complication post PEG-J insertion. BBS has significant morbidity and even mortality in rare instances, warranting appropriate follow-up after these delivery systems are inserted and adequate post-insertion site care. The diagnosis of BBS in a PEG-J system can be significantly delayed when only the J extension is being used, but not the G port. Thus, we recommend frequent checks on the PEG-J system post placement to assure patency of the system by twice daily water flushes, in addition, follow-up should ensure that the external bumper is at least 1 cm from the skin. Removal of the PEG-J when BBS occurs can be safely performed in various techniques, with limited morbidity associated with successful replacement when the system is still indicated.
